# Recent Advancements in Materials and Coatings for Biomedical Implants

**DOI:** 10.3390/gels8050323

**Published:** 2022-05-21

**Authors:** Kamalan Kirubaharan Amirtharaj Mosas, Ashok Raja Chandrasekar, Arish Dasan, Amirhossein Pakseresht, Dušan Galusek

**Affiliations:** 1 Centre for Functional and Surface-Functionalized Glass, Alexander Dubcek University of Trencín, 911 50 Trencín, Slovakia; arish.dasan@tnuni.sk (A.D.); amir.pakseresht@tnuni.sk (A.P.); 2 Joint Glass Centre of the IIC SAS, TnUAD, and FChFT STU, FunGlass, Alexander Dubcek University of Trencín, 911 50 Trencín, Slovakia

**Keywords:** bioceramics, metallic implants, biomedical applications, coatings, surface modification, biocompatibility, Mg-alloys, Ti-alloys

## Abstract

Metallic materials such as stainless steel (SS), titanium (Ti), magnesium (Mg) alloys, and cobalt-chromium (Co-Cr) alloys are widely used as biomaterials for implant applications. Metallic implants sometimes fail in surgeries due to inadequate biocompatibility, faster degradation rate (Mg-based alloys), inflammatory response, infections, inertness (SS, Ti, and Co-Cr alloys), lower corrosion resistance, elastic modulus mismatch, excessive wear, and shielding stress. Therefore, to address this problem, it is necessary to develop a method to improve the biofunctionalization of metallic implant surfaces by changing the materials’ surface and morphology without altering the mechanical properties of metallic implants. Among various methods, surface modification on metallic surfaces by applying coatings is an effective way to improve implant material performance. In this review, we discuss the recent developments in ceramics, polymers, and metallic materials used for implant applications. Their biocompatibility is also discussed. The recent trends in coatings for biomedical implants, applications, and their future directions were also discussed in detail.

## 1. Introduction

Bioimplants are defined as engineered medical devices that are developed to replace the non-functional or broken biological structural parts of the human body, providing support to the given host. Biomaterial surface modification plays a key role in determining the outcome of the interaction between human biology and materials. Substantial development in research in the field of biomaterials has increased the scope of use for a wide range of orthopedic and dental implants that include total bone replacement, fracture fixation, dental screws, joint arthrodesis, and so on [1]. Essentially, the success of bioimplants depends not only on their bulk properties but also on the properties of their surfaces, which interact with human body tissues. As a result, the evolution of bioimplants has reached a level of choice of materials based on specific properties on the basis of selected specific materials [2]. Though alloys and metallic substances meet many of the biomedical requirements, their interfacial bonding between the surrounding tissue or bone and the metallic surface ranges from poor to virtually absent. The failure of the metallic implant originates at the implant-tissue interface due to poor bonding at the interface, which leads to the formation of a nonadherent layer and movement at the tissue-implant interface [3].

Corrosion in biometallic implants can affect the surface and biocompatible behavior that induce tissue reactions, which lead to the release of corrosion byproducts from the implant surface and result in premature failure. A minimum durability of 15 to 20 years for older patients and more than 20 years for younger patients is expected from a bioimplant [4]. However, there are problems associated with the use of metallic implants due to the lack of poor implant fixation, lack of osteoconductivity, corrosion, and wear resistance leading to the formation of wear debris and release of corrosive ions [5,6,7]. These problems are mostly associated with the surface of the metallic implants. In view of this, the surface of the bioimplant plays a major role in the biological environment because the reactions occur directly on the surface of the implant after implant fixation. Hence, it is necessary to modify the surface of the metallic substrate with specific properties that are different from those in bulk [8,9]. This modification is required to accomplish good bone formability and desired biological interactions. In some applications, biocompatibility, wear, and corrosion resistance are also required.

Surface modifications of bioimplants are explored intensively with many bioactive materials to avoid adverse effects such as lack of biocompatibility, post-surgery infections, long-term survivability, and risks related to implant surface corrosion [10,11]. At first, the research in this field was focused on the improvement in biomechanical properties of metallic implants, but in recent days, it has turned towards improvement in the biological properties of these biomedical devices [12,13]. By applying the appropriate modification on the surface of the material, one can tailor and improve the biocompatibility, cell interactions, and adhesion [14]. Thus, the development and design of biomaterials rely on surface modification. For that, it is necessary to develop techniques for functionalization of the surface of metallic implants through changing the materials’ surface composition, morphology, and structure without losing their mechanical properties. By adopting this, the service life and performance of orthopedic and dental implants can be significantly increased. This can be achieved by applying suitable biocompatible coatings with a unique combination of properties.

In view of reliability and performance, the best way to functionalize the implants in direct contact with bones and tissues is ceramic coatings owing to their excellent osteoconductive properties and high stability [15,16]. Surface modification by coating can enhance the antibacterial activity of a bioimplant. The coated surfaces facilitate grafting of cell-binding peptides, directed mutations of the cellular host, protein of extracellular matrix (ECM), and growth of tissues to improve the acceptance of a bioimplant further. Ceramic coatings on bioimplants show promising results in orthopedics with improved bone regeneration and repair [17]. The overview of applications of ceramic coatings used for metallic implants is listed in Table 1.

The major requirements for the selection of coating materials are (a) biocompatibility and nondetrimental effects such as allergy, inflammation, and toxicity, (b) adequate fracture toughness, fatigue, and mechanical strength to withstand the forces, and (c) resistance to corrosion in the human body fluid atmosphere, which contains many constituents such as amino acids, chlorine, water, proteins, sodium, and plasma acids. The choice of coatings, by considering their degeneration and surface properties, plays a major role in terms of reliability and performance of bioimplants. The coatings for biomedical applications can be subdivided into three groups: (a) bioinert, (b) bioactive, and (c) bioresorbable coatings [19]. The coatings having a minimum interaction with the surrounding tissues after implantation in the human body are considered as bioinert coatings. The typical examples of bioinert coatings are metal oxides, nitrides, carbides, carbonitrides, and oxynitrides. Transition metal nitrides (TiN, ZrN, TiAlN, NbN), carbides (TiC), oxides (ZrO_2_, Al_2_O_3_, TiO_2_), or oxynitride (TiON) coatings find a wide range of applications in bioceramic coatings due to their remarkable properties such as wear, tear, hardness, biocompatibility, and corrosion resistance [20,21].

The current review incorporates a description of the biomaterials and coatings that are commonly used in the manufacturing of different orthopedic and dental implants.

## 2. Biomaterials for Biomedical Applications

Biomaterials are used to make devices that interact with the biological systems in the human body and coexist for a long time with minimal failure. The type of material used in implant applications shows specific properties that make them primary candidates for specific applications. The key requirements for the selection of biometallic materials consist of (a) cost effectiveness, (b) mechanical behavior equal to that of the human skull and bones, and (c) their biocompatibility [22,23]. In addition, the major requirement for the bioimplant materials is that it should be compatible with the human body, i.e., it should integrate with the human body without negative impacts. Moreover, it must possess corrosion and wear resistance in the human body environment. These properties will determine the effectiveness of the implant materials.

If a metallic material experiences wear and corrosion, the surrounding tissues present at the implant area can become inflamed, causing unfavorable biological reactions within the human body [24]. The ions and toxins released from the metallic substrates as a byproduct may be potentially harmful and can cause life threatening diseases and increase the risk of using metallic implants. Therefore, it is important to choose correct material for correct applications while performing bioimplants. In addition to that, the mechanical performance of the biomaterial should be close to that of the replacing material where it must sustain complicated and varying mechanical loading cycles [25]. Typical examples for implanting areas are teeth, knee joints, and hips. The selection of biomaterial based on mechanical properties is important to ensure no implant failures within the body when subjected to numerous loading cycles during service life. Moreover, the material should be biocompatible with the surrounding tissues and economically viable. Finally, it is essential that the choice of material should be cost effective, efficient, and able to integrate with the human body. Based on the requirements defined above, several materials were developed in recent years to be used as biomaterials for implant applications. Still, it is hard for a single metallic material to fulfill the desired properties. Biomaterials used for biomedical applications are broadly classified into ceramics, polymers, and metallic systems.

### 2.1. Ceramics

Ceramics are inorganic compounds formed at high temperatures. Typical examples are bioactive glass (BG), zirconium oxide (ZrO_2_), aluminum oxide (Al_2_O_3_), hydroxyapatite (HAp), and other calcium and silica-based ceramics. These ceramics are noted for their great biocompatibility, which makes them an excellent candidate for biomedical implant applications. Depending on the reactivity with the human body, ceramic implants are classified into three categories: (a) bioactive, (b) bioinert, and (c) bioresorbable ceramics [26]. Bioactive ceramics are used to interact with the surrounding cells and exhibit a higher level of reactivity within the implant sites. Typical examples for bioactive ceramics are HA and fluorapatites [27]. In an opposite trend, bioinert ceramics do not show any reactivity with the host tissues at the implant sites but form a physical bonding when implanted [28]. Bioresorbable ceramics exhibit a low level of reactivity with the host body tissues [29]. After implantation, these ceramics are gradually resorbed and finally replaced with the bone tissue. These bioresorbable ceramics are widely used in orthopedics and dentistry due to their better biocompatibility and chemical interactions [30].

No risk of transmitting disease plus immunogenicity after implantation are the major advantages of the ceramics [31]. Other remarkable advantages are higher resistance to compressive force, low toxicity, good corrosion resistance, and promotion of the formation of new hard tissues. For example, hydroxyapatite-based ceramics exhibit higher Ca/P ratios, which are desirable due to similar chemical properties of bone and teeth hard tissues [32,33,34,35]. Due to these attractive properties, ceramics are increasingly utilized for bioimplant applications.

Ceramics are known for their high hardness and stress-shielding effects due to their high elastic moduli, and slow initiation of crack growth over time, which significantly decrease the reliability of the implants [36]. In addition, brittleness, fracture toughness, and fabrication issues limit their use as bioimplants. The ceramics share the brittleness factor, which limits the performance in terms of load-bearing applications (hip implants). If the difference in mechanical properties of ceramic and bone is large, the load will not be transmitted through the bone, thus leading to failure of the bone [37].

Ceramic composite materials provide superior properties compared to single materials. The inferior mechanical properties of monolithic ceramics can be overcome by composite ceramics while diminishing the limitations of each component. The remarkable properties of composites such as the weight to strength ratio enable them to be used extensively for the restoration of bones, ligaments, and dental fillings [38]. Moreover, the composites prepared through the combination of bioactive and bioinert ceramics show better bioactivity and mechanical strength [39]. Typical examples are HA and Al_2_O_3_ composites which show better osteointegration with bone, good bioactivity, and high yield strength [40,41].

### 2.2. Polymers

The most widely used materials in biomedical applications are polymers. Polymers are the building blocks of small repeating units’ monomers and are classified into two categories called biodegradable and non-biodegradable. Typical examples for biodegradable polymers are polyacetal, chitosan (CS), alginate, polylactide, and polycaprolactone, whereas non-biodegradable polymers include polypropylene, polytetrafluoroethylene, polyethylene terephthalate, polymethylmethacrylate, etc. Polymer implants are mostly used in replacing heart valves, kidneys, bone, skin, contact lens, and artificial blood vessels, in addition as pacemakers [42]. Among biodegradable polymers, CS shows remarkable properties such as biocompatibility, biodegradability, wound healing, and antibacterial activity [43]. It is also environmentally friendly and hence acts as a capping agent [44,45]. Polymers show lower strength and elastic moduli as compared to metals and ceramics. Therefore, they are not generally used for load-bearing applications such as joint and knee prostheses. The polymers are also degraded in the body environment due to biochemical factors.

Polymer implants are quite interesting as bioimplants due to their low cost while offering sufficient mechanical properties. For example, Polyether ether ketone (PEEK), composed of 20% TiO_2_ particles and an additional ketone group results in 80% higher compressive strength and better fatigue properties than pure PEEK [46]. Depending upon the replacement anatomy to which the polymer is being applied, a wide variety of polymers can be applied. Polymers have the advantage of complete degradation over time, leaving no signs of their presence at the implant locations in a body. This was possible with the subsequent research and development in biodegradable polymer materials, where the proteins and extracellular matrix mimic the cell signaling functions of the surrounding tissue, permitting better bio-integration [47].

Though polymers show exceptional properties and are cost-efficient and easy to manufacture, they show different forms of cytotoxicity: depending on the host body conditions, inflammatory reactions can occur within the implant region. This will induce bone degeneration, abnormalities, rapid rate of corrosion, and decreases in mechanical properties over time. Moreover, the elastic modulus of polymers is extremely low compared to human bone (between 10 and 30 GPa) [48]. This will create an impact while applying load. Another major issue that is being faced is that the polymer implant degrades as the bone heals. If the process is too fast, the neighboring tissues feel more stress, which causes potential discomfort. These limitations prevent them from being widely used as bioimplants.

#### Polymeric Gels

Natural polymers such as collagen are the main components of natural bone due to their hydrophilic nature, enabling the formation of hydrogels with aqueous solutions that exhibit several desirable characteristics for bone-tissue engineering [49]. Polymeric gels are often referred to as hydrogels owing to their ability to hold water inside their networks [50]. These hydrogels swell upon water intake and shrink upon drying [51]. Taking advantage of this property, water soluble drugs, growth factors, and other biological entities such as proteins and even live cells can be incorporated into these hydrogels [52]. These gels can be designed for delivery systems based on certain external stimuli such as pH [53,54], temperature, or the presence of specific chemicals or target molecules [55]. Many researchers choose collagen because it is the most important organic component of human bone [56,57,58].

Hydrogels are attractive soft biomaterials because of their soft consistency (stiffness and viscoelasticity are essential in directing the immune response), high water content, porosity, and biocompatibility [59]. They are widely used in 3D cell cultures for modeling the biological extracellular matrix or as coatings for promoting cell attachment. Other natural polymer-based hydrogels used as bone tissue engineering (BTE) materials include polysaccharides (e.g., cellulose) and polypeptides (e.g., alginate). Compared with natural polymeric gels, synthetic polymeric gels offer more possibilities for molecular alterations that facilitate tailoring the candidate properties to specific requirements, i.e., tuning mechanical properties and biophysical and biochemical cues. For instance, Poly(ethylene glycol) (PEG) hydrogels, modified with adhesion ligand arginine–glycine–aspartic acid (RGD), offer tunable mechanical properties as well as improved cell attachment and cell differentiation [60]. However, generally, the poor mechanical strength of hydrogels limits their usage and needs further improvement for bone regeneration. Recent emerging technologies such as 3D printing in the manufacturing of hydrogel-based components may offer entirely new possibilities for addressing the challenges [61].

### 2.3. Metals and Alloys

Even though ceramics show excellent biocompatible performance, they have poor fracture toughness and exhibit brittle behavior, and their use in load-bearing applications is limited. Thus, metals and alloys are generally used for implants where high strength and load-bearing capacity are required. Most medical industrial segments rely on metallic implants. They are generally used to replace some load-bearing applications such as the hip, plates, knee prostheses, pins, dental materials, screws, and cardiovascular applications [62]. Though metals show high strength and durability, they can lose their properties under physiological conditions with a potential release of various ions and debris which may trigger a biological response. Most of the alloys release metal ions to the plasma in the blood [63]. The excessive release of ions in the blood has a high risk of accumulation in organs such as the spleen and liver that later form particulates, affecting the normal functioning of these organs. This phenomenon leads to cytotoxicity followed by organ failure upon prolonged accumulation.

Metallic materials are not fully accepted by the human body, and the tissue growth is impaired because of inadequate attachment of the implant, leading to discomfort or pain in the implant region [64]. As compared to ceramic materials, the risk of infection is higher, and the healing time is slower in the case of metallic implants. Although metallic implants have some limitations, preference should be given based on their corrosion resistance, cost effectiveness, and mechanical strength. The chemically inert platinum and gold do not show any corrosion in situ, and these materials can be used as bioimplants, but they are expensive. Hence, recent biomedical industries use Ti-based alloys and Mg-based alloys due to their better biocompatibility and good mechanical strength under human body conditions [65]. The widely used metallic materials used as biomedical devices are stainless steel and Ti- and Co-based alloys [66,67].

#### 2.3.1. Stainless Steels (SS)

In India, SS 304 and 316L are the most used implant materials for biomedical applications due to their cost effectiveness, wide resource availability, reliability, and ease of fabrication as compared to Ti- and Co-based alloys. Among various grades of SS, the primary recommended grade for implant applications is AISI type 316L SS. The presence of chromium (minimum content of 10.5 wt. %) yields a thin and passive oxide layer and protects the implant surface against corrosion [68]. The presence of carbon (min. 0.03 wt. %) in SS increases its mechanical properties, especially fracture toughness, corrosion resistance, and tribological performance of the implants. Their load-bearing capability makes them a suitable orthopedic implant material [69]. However, almost 90% of 316L grade SS implants lose their properties due to a pitting corrosion attack and the release of nickel and chromium ions, which cause allergic reactions in the implant region. Hence, a small addition of molybdenum (2 to 4 wt. %) improves the corrosion resistance and strengthens the 316L SS grade.

The 316L SS used in biomedical devices is classified into two categories: (a) conventional SS and (b) Ni-free stainless steels [70]. The primary use of conventional stainless steels is to provide a load-bearing property to the implanted surfaces: they are often used as fracture plates, nails, screws, and stents in the implant process. In addition, the Ni-free SS provides higher corrosion resistance and biocompatibility [71]. When compared to other bioimplants, the chemical composition of SS alloys offers an advantage when good mechanical properties are desired. Moreover, they have a high cost-to-benefit ratio and exhibit a linear relationship with the manufacturing processes and final structure/properties.

Its elastic modulus (200 GPa), which is higher than that of the human bone (10–30 GPa), results in high stress-shielding effect at the tissue/implant interface leading to the failure of the implanted SS [72,73,74]. In recent days, SS was modified with hydroxyapatite (HAp) which improves its bio-integration and osteointegration properties. Typical implanted materials are screws, pins, sutures, bone plates, steel threads, and medullary nails, which are used in fracture fixation. However, the corrosion resistance, biocompatibility, and osseointegration of SS are lower compared to Ti-based alloys, where implant success rates are much higher [75].

#### 2.3.2. Co-Cr Alloys

Co-based alloys are considered as one of the most successful materials used for implant applications. This alloy was first used in the early 1900s, where it was used as an implant material for hip replacement. Co-based alloys show better corrosion, wear, and mechanical properties and are used in bioimplant applications. The in vivo and in vitro studies confirmed that Co-based alloys show better biocompatibility and can be used for the manufacturing of surgical implants such as in the hip, knee, shoulder, and fractured bone surfaces [76,77]. The most widely used combination of Co alloys are Co-Cr-Mo owing to their unique combination of strength and ductility. By comparing with other metallic implants, this alloy shows a better elastic modulus, density as well as stiffness, becoming an ideal material for the implant process [78]. This alloy is primarily focused on permanent implant fixation procedures because these alloys maintain their initial properties for a long time after implantation. The cumulative likelihood of endurance reached 96% at 12 years for patients aged above 60 years [79]. A Co-Cr-Mo alloy combined with ultra-high molecular weight polyethylene (UHMWPE) is used in artificial ankles and knees [80,81].

Other major alloying elements of Co-based alloys include Ni, Mo, and Cr. These elements were proven to be toxic to the human body when leached out from the metal surface to the body fluid during corrosion of Co alloys and can lead to skin-related diseases. An excessive leaching of these trace elements leads to damage to organs such as the liver, kidney, blood cells, and lungs [82,83]. The addition of nickel into Co-Cr-Mo improves corrosion resistance and mechanical properties, but due to the cytotoxicity of Ni, the use of this alloy in bioimplants is limited [84]. The elastic modulus (200–250 GPa) and ultimate tensile strength (400–1000 GPa) of Co-based alloys are 10 times higher than those of the human bone. The use of these implants manufactured from Co-based alloys thus results in a stress-shielding effect at the tissue/implant interface. The surface modification of Co-Cr-Mo alloys under plasma treatment improves hardness, wear, and corrosion resistance [85,86,87]. However, they are still not recommended for joint fixtures due to their inferior frictional and tensile properties. Apart from their biocompatibility and corrosion behavior, Co-based alloys are not ideal materials for bearing and joint surfaces due to their sub-par frictional properties [88].

#### 2.3.3. Ti Alloys

Commercially pure titanium (Ti) and its alloys (Ti-6Al-4V, Ti-6Al-7Nb, Ti-5Al-6Nb, and Ti-13Nb-13Zr) have become major assets in the biomedical field owing to their superior biocompatibility, low density, and suitable mechanical properties. At first, it was intended to be used for aerospace applications, but later in the 1970s, the discovery of its biocompatibility led to a demand for Ti and Ti alloys in biomedical applications. If commercial pure titanium (Cp Ti) is used to replace its alloys, the mechanical properties lost due to alloying elements must be compensated for [89,90]. The alloys of Ti show enhanced mechanical and biocompatibility properties in comparison to pure titanium. Depending on the presence of the iron and oxygen content in the Ti alloy, four different grades of alloys are used. The most widely used Ti alloy is Ti-6Al-4V, comprising an estimated 50% of total titanium alloys’ usage for bioimplants of this grade [91,92]. By comparing with other grades of Ti alloys, it offers excellent corrosion resistance, biocompatibility, formability, structural stability, and a better weight to strength ratio. The applications of Ti alloys as bioimplants include heart valves, dental prostheses, osteosynthesis, artificial joints, and bone replacements [93].

Biomedical grade titanium alloys are generally categorized as alpha (α, Ti-6Al-4V), near-α, α-β, and metastable β (Ti-6Al-7Nb) [94,95]. These alloys are widely used as biometallic implants, but they cause stress shielding issues at the implant-tissue interface due to their high elastic modulus values. The elastic modulus of Ti and α-β Ti-alloys (100–110 GPa) is higher than that of human bone which limits its usage in joints. The presence of vanadium and aluminum compounds results in the release of toxic ions of vanadium (oxidovanadium (IV) and vanadate (V)) and aluminum (Al^3+^) under the physiological environment, leading to adverse health issues [96,97,98]. Therefore, much interest has been paid to β alloys in combination with Zr, Nb, Ta, or Mo to replace V and Al in the alloy. Such alloys possess better mechanical properties, ductility, good structural stability, higher wear resistance, a lower elastic modulus, and improved corrosion resistance [99,100,101].

One of the disadvantages of using Ti alloys is their below par tribological properties, due to their high friction and abrasive wear nature [102,103]. Moreover, the formation of TiO_2_ during exposure protects the surface of the Ti alloy, which hinders the bioimplant-tissue relationship. The formation of titanium compounds around the surrounding tissues of the implant causes failure of the implant [104].

#### 2.3.4. Mg Alloys

Metal-based biodegradable orthopedic implants nullify the complications associated with the long-term existence of implants inside the human body. In recent days, biodegradable metallic implants were investigated as biomedical implants [105]. Magnesium (Mg) is present in the human body as the fourth most abundant cation and is essential to the human metabolism. Mg corrodes faster in the chloride containing physiological environment; thus, it has emerged as biocompatible and biodegradable material for use as implants [106]. Moreover, Mg and its alloys have received much attention in the category of biodegradable alloys due to their leading properties such as low density, an elastic modulus close to that of bones, light weight, biocompatibility, and excellent mechanical properties [107,108]. The revision surgeries performed to remove hardware components in implants such as screws and plates from the implanted site after healing are often discomforting and expensive for the patients. The revision surgery can also lead to complications such as nosocomial infection and delay the patient’s recovery to a normal lifestyle. Mg-based biodegradable metallic implant components can overcome the revision surgery by degrading in situ, thus also eliminating the need for the procedure to remove the implant components after healing [109].

The high mechanical strength of metallic materials limits the use as bioimplants, whereas the Mg implant shows a reduced elastic modulus and prevents the mismatch between a bone and the Mg-based implant. This leads to the reduction in stress shielding at the bone/implant interface. Their mechanical and corrosion properties can be enhanced by alloying with Al, Zn, and other elements [110,111]. Current research is focused on the development of Mg-based alloys with zero or low cytotoxicity. Alloying Mg with other metals must be selected carefully to avoid metal-related toxic issues and corrosion. Different type grades of Mg alloys such as Mg-Ca and Mg-Y-Nd were studied as biodegradable bioimplants for orthopedic applications [112].

The major limitation associated with Mg and Mg-based alloys is their rapid corrosion in physiological conditions. Rapid corrosion results in quick release of byproducts such as hydrogen gases due to fast in vivo degradation. This indicates the necessity for surface modification. To overcome the rapid corrosion, alloying with various elements has been explored. For example, elements such as calcium (Ca), zinc (Zn), silver (Ag), aluminum (Al), zirconium (Zr), yttrium (Y), and Neodymium (Nd) were added to Mg to enhance the corrosion and mechanical properties [113,114,115,116,117]. Typical examples are Mg-Ca, Mg-Zn, and Mg-Zn-Ca. By carefully selecting a suitable element and its composition, the microstructure can be tailored to meet mechanical properties such as bone. This makes them ideal for bone replacement. Table 2 shows the overall comparison of materials used for biomedical applications and their applications.

## 3. Need for Surface Modification of Bioimplants

**Figure 1:**
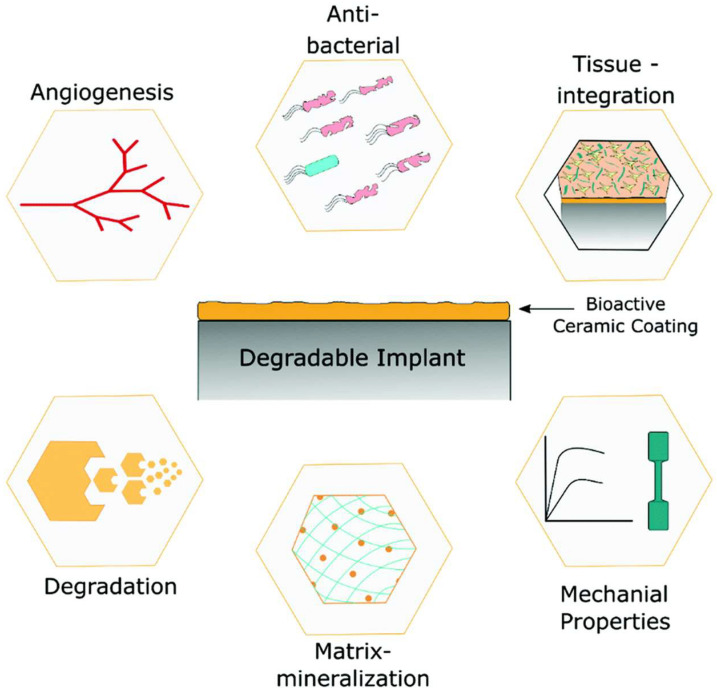
The role of bioactive coated metallic implants as a potential implant material [127]. The qualities of coated implants are superior to those of uncoated metallic implants.

In an implant operation, any material inserted into the human body is treated as a foreign substance. If the foreign substances are not biocompatible, layers of fibrous tissues, also known as scar tissues, begin to develop between the tissue and implant. Eventually, due to scar tissue development, the implant fails to osteointegrate with the host bone, leading to implant failure. Therefore, the primary requirement for the successful implant process is to have a complete integration between bioimplants and human body tissues [120]. The biological responses of biomedical devices to the lifespan and performance are better controlled by their surface morphology and chemistry. To achieve better biocompatibility and osteoconductivity, surface modification on biometallic materials has been recommended to achieve the desired properties (Figure 1) to increase the success rate of implants. When the surface is effectively modified, the bulk functionality and properties of the biomedical implant device will remain unaffected for a long time [121,122]. With the advantage of bio-integration and the load-bearing capability of biomaterials, the success rate for bioimplants can be greatly increased.

In recent years, researchers tried to enhance the bio-integration of implants by modifying the implant surface that is in contact with the body environment. Two approaches are considered for modifying the surface of the implants. The first approach is to deposit organic/inorganic-based coatings on the metallic surface without modifying the implant substrate [123]. The second approach is to use conversion coatings or surface modified layers, where the chemical surface modification of a substrate results in a slight increase in thickness [124]. In this case, the substrate elements are involved in developing conversion coatings. For conversion coating, surface preparation by grinding and polishing is required to improve the surface roughness for better mechanical interlocking of coatings. This process is critical, and surface modification by depositing an overlay coating is recommended [125]. Recently, a combination of both surface modification and deposition of thin films was performed to achieve the synergy of both properties.

In a modern biomedical implant industry, surface modification of metallic implants with an appropriate coating material is used to enhance biocompatibility, corrosion resistance, antimicrobial behavior, and mechanical properties. Although there are many methods for the deposition of bioactive surface coatings, an optimal coating technique for biomedical applications has not been developed yet. Currently, the coatings on implant materials are deposited by one of the deposition techniques such as physical vapor deposition (PVD), chemical vapor deposition (CVD), electrophoretic deposition (EPD), electrodeposition (ED), or sol-gel methods [2]. Among these, PVD is recommended to deposit metal/ceramic materials over the implant surface and provide exact stoichiometry, excellent adhesion, high density, and good uniformity. Another method for surface modification other than coating methods is chemical etching to prevent bacterial adhesion and improve osseointegration [126].

The success of an implant is dependent on the stability of the coating, which provides better biocompatibility. This section is focused on the recent advancements in various types of ceramic and polymer coatings to improve bioimplant performance and reliability.

### 3.1. Polyether Ether Ketone (PEEK)

PEEK is a thermoplastic material that shows a combination of excellent stiffness, chemical and physical properties, and toughness and offers a wide range of applications [128]. Therefore, it is widely used as a bone substitute in orthopedic and dental implants, and in clamps for removable dental prostheses [129]. The PEEK coated substrates show better tribological properties, which are useful for the development of coatings on light weight alloys which lack tribological performance. Most of the sliding and bearing implant materials are coated with PEEK due to its better wear resistance and thermal stability [130,131]. Generally, PEEK coating and its composites are prepared using thermal spraying or electrophoretic processes [132,133,134,135]. PEEK coating (70–90 µm thick) deposited through electrophoretic deposition on the Ti-13Nb-13Zr titanium alloy showed excellent wear resistance, 200 times higher than the uncoated alloy [136].

PEEK in combination with other bioactive materials shows better antibacterial activity than PEEK alone [137]. Many authors reported on PEEK-based composite coatings on metallic substrates. These coatings enhance bioactivity and electrochemical corrosion resistance, especially for implant structural components. Typical examples for the composite coatings are TiO_2_/PEEK [138], sol-gel glass/PEEK [139], bioactive glass/PEEK [140], h-BN/PEEK [141], Ag/bioactive glass/PEEK [142], and h-BN/bioactive glass/PEEK coatings [137]. A combination of bioactive glass embedded in a polymeric matrix of PEEK makes it an interesting material for orthopedic applications as it meets biological and biomechanical requirements for the application. A cold sprayed Bioglass/PEEK composite prepared by Garrido et al. [143] showed an increase in wear resistance by more than 70%, higher hardness, and a lower coefficient of friction compared to pure PEEK. Coatings based on Bioglass/PEEK on porous Ti substrates resulted in higher adhesion between Bioglass/PEEK coating and Ti substrates [144].

Flame sprayed hexagonal boron nitride (h-BN) incorporated PEEK coating on low-carbon steel substrate increased the hardness and decreased wear and frictional coefficient values for the composite coating containing 8 wt. % h-BN due to its self-lubrication properties [145]. The coefficient of the friction value can also be reduced by the addition of alumina. The Al_2_O_3_/PEEK composite coating deposited on a Ti alloy using electrophoretic deposition showed increased corrosion resistance and significantly improved wear resistance under dry sliding conditions. The viability test revealed that the Al_2_O_3_/PEEK coating was found to be cytocompatible with MG-63 osteoblast cells [146]. The scratch resistance of PEEK coatings can be increased with the addition of amorphous Si_3_N_4_ nanoparticles. Tomasz et al. [147] performed the electrophoretic deposition of the PEEK/Si_3_N_4_ nanocomposite using a chitosan stabilizer: the coating showed higher scratch resistance than PEEK coating alone. This suggests that PEEK-based nanocomposite coatings potentially improve the bioactive as well as bio-tribological performance of Ti-based alloys used in biomedical applications. The use of PEEK with HAp as a coating can reduce the stress shielding effect. The combination of PEEK/HAp offers similar stiffness to that of the bone tissue. Recent studies suggest that the incorporation of HAp into PEEK coating improves bioactivity and mechanical properties [148]. PEEK coating prepared by different methods and their properties are summarized in Table 3.

### 3.2. Titanium Dioxide (TiO_2_)

TiO_2_ coatings are the most important materials in biomedical applications that are known for their antibacterial properties along with good mechanical properties. The applications of TiO_2_ coating include drug delivery systems [155], orthopedic [156], and dental applications [157]. It also shows high catalytic activity, antibacterial activity, and long-term stability under photo and chemical corrosion [158]. TiO_2_ promotes the formation of bone-like apatite or calcium phosphate on its surface. This property makes it a suitable candidate for reconstruction and bone replacement [159].

TiO_2_ coated metallic substrates show better antibacterial properties. Gartner et al. [160] observed the same biocidal effect by applying TiO_2_ coating on glass substrates by a sol-gel method. Photocatalytic activity of TiO_2_ coating received much attention as a potential material for anti-bacterial coatings. The antibacterial effects of TiO_2_ coating involve both a reduction in bacteria’s viability and their destruction [161]. Park et al. [162] showed that the antibacterial effect against *S. aureus* could be improved by adjusting the nucleation time of TiO_2_ film during the deposition process. The antibacterial effect of TiO_2_ was explained by the formation of reactive oxygen species. Apart from antibacterial properties, the antiviral properties of the TiO_2_ coating are also studied [163]. Table 4 summarizes the use of TiO_2_ and its composite coatings for bioimplant applications.

Yetim [164] prepared TiO_2_ coating with different concentrations of Ag using the sol-gel process on the commercially pure titanium substrate. Electrochemical corrosion properties obtained from electrochemical impedance spectroscopy measurements and potentiodynamic polarization tests in simulated body fluid (SBF) suggest that Ag doped TiO_2_ enhances corrosion resistance over that of the bare Ti substrates as well as undoped TiO_2_ coated samples [165]. The silver doped TiO_2_ (Ag/TiO_2_) nanocomposite coated glass substrate with varying Ag content synthesized by the sol-gel route showed antiviral properties against *E. coli*, enterovirus, and influenza A virus (H1N1) [166]. The highest level of photocatalytic degradation under irradiation with either visible or ultraviolet light was observed at an optimum Ag:TiO_2_ weight ratio of 1:100. The antibacterial effectiveness was greater than 99.99% against *E. coli* and other infectious diseases after visible light illumination.

**Figure 2:**
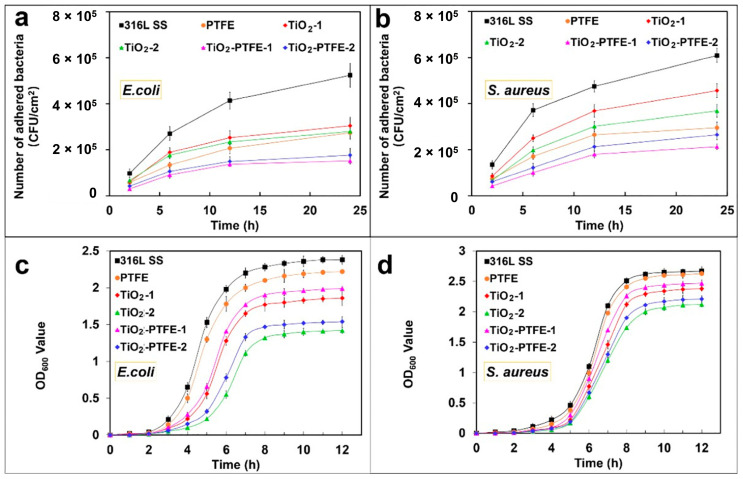
Effect of bacterial adhesion (**a**,**b**) and bacterial growth of *E. coli* and *S. aureus* pathogens on TiO_2_-PTFE coated and uncoated substrates [164]. TiO_2_-PTFE coated substrates exhibit lower bacterial adherence and a significant reduction in bacterial growth (**c**,**d**) as compared to uncoated substrates.

Sol-gel derived TiO_2_-PTFE nanocomposite coating on stainless steel substrates was prepared by Zhang et al. [164] and their bacterial adherence were tested against two pathogens, namely *S. aureus* and *E. coli*. The bacterial adhesion and bacterial growth studies were evaluated by fluorescence microscopy after 2 h, 6 h, 12 h, and 24 h of incubation (Figure 2a,b). The TiO_2_-PTFE coated substrate shows the lowest bacterial adhesion when compared with the uncoated substrate. The bacterial inhibition increases with the increasing TiO_2_ concentration (Figure 2c,d). It is also observed that Gram-positive bacteria are less sensitive due to their cell wall thickness.

**Table 4:** Uses of TiO_2_ and its composite coatings in bioimplant applications.

S. No.	Coatings	Deposition Method	Significance	Ref.
1	TiO_2_ coating on Ti substrates	Anodic oxidation	Potential rehabilitation to internal bone fracture	[167]
2	TiO_2_ coating on PEEK substrate	Dip coating	Recommended for maxillofacial and oral implants applications	[168]
3	TiO_2_/MoSe_2_/chitosan coating on Ti implants	Micro-arc oxidation process	Excellent in vivo and in vitro antibacterial property against *S. mutans* Better biocompatibility and hydrophilicity Better antibacterial properties	[169]
4	Poly(epsilon-caprolactone)/titania (PCL/TiO_2_) coating on Ti implants	Electrospinning technique	Good bioactivity against osteoblast cell Superior antibacterial against *S. aureus* Promoting cell attachment	[170]
5	TiO_2_ coating on Ti substrates	Direct lithographic anodic oxidation	Corrosion resistant	[171]
6	TiO_2_ nano coating	Anodizing oxidation technique	Better cell proliferation and adhesion Better osseointegration	[172]
7	Graphene/TiO_2_ coating on Ti substrate	Drop casting method	Better cell adhesion and proliferation behavior	[173]
8	TiO_2_/HAp bilayer coating on Ti substrate	MOCVD/Plasma spraying	Better hardness In vitro bioactivity	[174]
9	Y-doped TiO_2_ coating on Ti alloy	Plasma electrolytic oxidation method	Better antibacterial activity against *E. coli* and *S. aureus*	[175]
10	Fe_3_O_4_/TiO_2_ composite coating on Ti implants	Micro-arc oxidation process	Prevent inflammatory Better fibroblast response	[176]

### 3.3. Transition Metal Nitrides

Earlier, transition metal nitrides and carbides were widely used to protect the metallic components against wear, tear, and corrosion, potentially offering high-temperature stability. Titanium nitride (TiN) coatings were used as decorative coatings in earlier days. In the last decade, nitride coatings for orthopedic implants were also proposed to protect the implants against wear and tear and to act as a diffusion barrier layer preventing the toxic ion release from the implant metal surfaces to the human body fluids [177,178,179,180]. The physical properties of TiN coated substrates show high scratch resistance, hardness, and low frictional coefficients. These properties make them a potential candidate for use as coatings on different metals used in arthroplasty. TiN-based coatings used for orthopedic applications show better biological properties as compared to other nitrides [181]. TiN coatings show better blood tolerability properties with a hemolysis percentage near zero [182]. TiAlN is another biocompatible nitride that has proven to be a promising alternative to TiN in biomedical applications despite its aluminum (Al) content [183].

Transition metal carbonitrides (TiCN, ZrCN) were found to increase the service life of orthopedic implants in terms of wear resistance in biological media [184,185,186]. Recently, quaternary carbonitrides-based coatings (TiAlCN, TiCrCN, TiNbCN, etc.) were found to show increased anticorrosive, mechanical, and tribological properties compared to ternary carbonitride-based coatings [187,188,189]. The tribological properties of these carbonitride coatings are very complex. However, the carbon-based carbonitride coatings show good biocompatibility, better wear resistance, and low friction [190]. Much attention has been paid to developing MeSiC-, MeSiCN-, and MeSiN- (where Me is a transition metal, and Si is an alloying element) based hard coatings [191,192,193,194]. These types of coatings show high thermal stability, a low frictional coefficient, excellent wear resistance, and good mechanical properties (hardness, Young’s modulus). Moreover, in many investigations, TiSi-based carbide and carbonitride coatings proved to be a potential candidate for a metallic implant which combines the mechanical, tribological, and anticorrosive properties of TiN and TiC with the biocompatibility behavior of SiC and SiCN [192,195,196,197].

TiN coating shows plastic deformation at the coating/surface interfaces due to dissimilarities in the hardness of the substrate and coating [198]. Thus, TiN coating cannot accommodate the fracture and deformation that creates flakes, and defects in the coatings cause deterioration of the coatings from the substrate. Therefore, chromium nitride (CrN) and chromium carbonitride (CrCN) coatings are recommended, which act as a better diffusion barrier for ion release from the alloys. These coatings also exhibit higher toughness, higher cohesive strength, and lower wear debris than TiN coatings [199].

**Figure 3:**
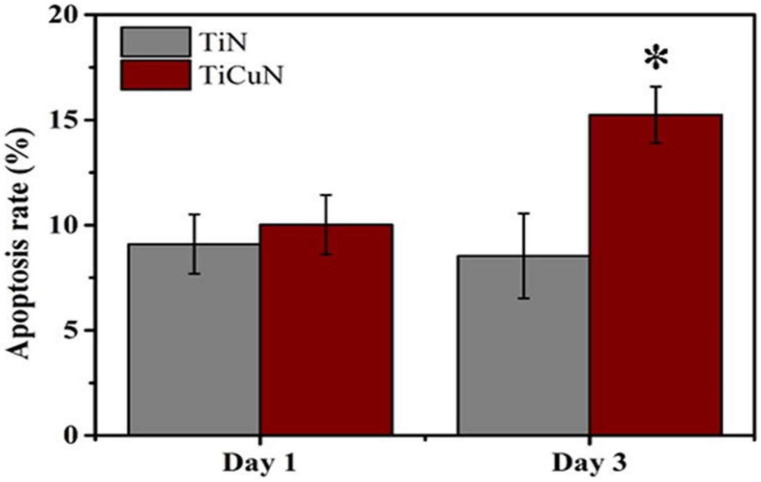
Apoptosis rate of TiN and TiCuN coatings tested for Day 1 and Day 3. Annexin V-FITC/PI double staining kit was used to evaluate the apoptosis rate of these coatings [200]. TiCuN coating promoted the early cell apoptosis rate more than TiN coating. *: Denotes TiCuN coating superior performance.

TiN and TiCuN coatings were prepared by the axial magnetic field enhanced arc ion plating (AMFE-AIP) technique, and the in vitro angiogenic response of human umbilical vein endothelial cells was studied by Liu et al. [200]. The TiCuN coating showed better antibacterial activity, and both coatings showed no cytotoxicity to human umbilical vein endothelial cells (HUVECs). TiCuN coatings promote early cell apoptosis, which is important for vascular tissue modeling (Figure 3).

Transition metal oxynitrides have been considered as interesting materials due to their known mechanical properties, chemical stability, and corrosion resistance in simulated body fluid. Zirconium oxynitride (ZrON) and titanium oxynitride (TiON) based coatings were recently used in biomedical applications for their better corrosion resistance than TiN coating and their anti-fouling ability [201,202]. The magnetron sputtered ZrON and TiON coated 316L SS specimen show better hardness and wear resistance behavior than the uncoated substrate [203]. In addition, both coatings show better anti-fouling performance against *Pseudomonas aeruginosa* bacterial adhesion than uncoated substrates. The coated substrates also show better corrosion protection with or without the addition of hydrogen peroxide (H_2_O_2_) in artificial blood plasma (ABP) solution [203].

Surface modified coatings prepared from ternary nitrides such as TiZrN, TiCrN, and TiAlN gained considerable attention because they retain their physiochemical properties, such as oxidation resistance, hardness, corrosion resistance, biocompatibility, and structural stability after implantation [204,205]. Magnetron sputtered TiZrN coated 316L SS substrates showed less bacterial adhesion, increased corrosion protection, and negligible human blood platelets activity than uncoated substrates [206]. Recent developments in binary, ternary, and quaternary systems of transition metal nitrides and carbide coatings are tabulated in Table 5.

### 3.4. Carbon Based Coatings

Carbon based materials are categorized under bioinert coatings. These coatings are used in load-bearing applications and wear components to improve elevated corrosion resistance, wear, and frictional effects [219]. Besides, carbon-based coatings show minimum protein adhesion and very good biocompatibility due to the hydrophobic nature of carbon-coated surfaces. Three different types of carbon-based coatings are used for biomedical applications. They are (a) nanocrystalline diamond (NCD), (b) pyrolytic carbon (PyC), and (c) diamond-like carbon (DLC) [220]. Some of the coatings are commercially available, while others are under development.

Most of the PyC coatings in biomedical applications are found in the heart valves due to their thromboresistant qualities and biocompatibility [221]. Most of the artificial heart valves are lined with a thick PyC coating. PyC biocompatibility in heart valves is well established. PyC coatings have also been used in orthopedic applications [222]. By varying the process parameters of the PyC (such as temperature, surface area, gas flow rate, precursor) in the CVD process, a variety of the structures can be produced. The most interesting structure for biomedical applications is lamellar, isotropic, granular, and columnar [223,224,225]. PyC coated orthopedic implants are used to replace small joints such as wrist joints, knuckles, and arthroplasty of proximal interphalangeal joints [226].

Carbon coatings, including nanocrystalline diamond and DLC coating, show many remarkable biological properties and are considered as coatings for medical implants. NCD coatings deposited by the CVD process consist of sp^3^-hybridized carbon bonds and show grain sizes in the range of a few nanometers. NCD coatings generally show very low surface roughness and possess the properties of a diamond, such as hydrophobicity and excellent biocompatibility with blood [227,228]. This makes them an ideal coating choice for wear-resistant implant applications and cardiovascular devices. NCD coating can also be used as hard antibacterial coatings that reduce the risk of infections. The electrically active NCD coating surfaces can establish a chemical bond with the biomolecules in the surrounding environment. Medina et al. [229] observed that the NCD coating surfaces react with the cell wall or membrane of Gram-negative *P. aeruginosa* bacteria and establish a chemical bond that alters the bacteria morphology, hindering bacterial adhesion and colonization on the surface of the coating. The properties of NCD films are utilized in biosensing and neurochemical sensing applications [230].

**Figure 4:**
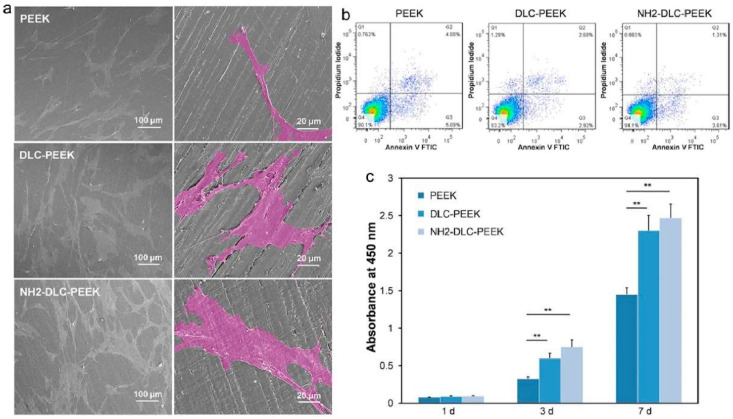
Represents in vitro cytocompatibility of DLC coated PEEK substrates, (**a**) surface morphology of hBMSC cultured on PEEK, DLC-PEEK, and NH2-DLC-PEEK substrates for 1 day, and the enlarged cells are shown in pseudo-color, (**b**) cell viability for 1 day, and (**c**) proliferation of hBMSCs after culturing samples for 1 d, 3 d, and 7 days [235]. ** denotes *p* < 0.01.

More experimental studies have been reported on DLC based coatings, which are considered as the most promising materials for bioimplant applications [231,232,233,234]. Medical grade PEEK samples were coated with DLC using plasma immersion ion implantation and deposition (PIII & D) technique, and their in vitro cytocompatibility and osteogenesis studies were carried out by Mo et al. using human bone marrow mesenchymal stem cells (hBMSCs) [235]. DLC coated substrates show better surface coverage of cells and show high cell viability on the seventh day, which indicates better biocompatibility of DLC-PEEK coatings than PEEK coating (Figure 4). However, DLC suffers from residual stress arising from the substrate/coating thermal expansion mismatch and lattice misfit, which cause poor substrate adhesion and delamination of the coating from the substrate. Another major concern about DLC coatings is their instability in the aqueous environment, which promotes delamination of the coating [236]. To avoid this issue, it is recommended to use interlayers (called buffer layer) such as CrC, Ti, and Si_3_N_4_ at the interface of the substrate and DLC coating [237]. Another approach is to dope DLC coating with N, F, Ag, Zr, or Ti to avoid a thermal expansion mismatch and residual stress [238].

The properties of DLC such as chemical inertness, surface smoothness, and hydrophobicity are important for providing better compatibility with blood, reducing platelet activation in contact with the blood, which could trigger thrombosis. DLC can act as a protective coating under the conditions of the human blood environment, which limits the release of nickel ions from metallic implants such as SS 316L. Several studies suggest that DLC coating prepared by various routes is biocompatible and does not induce any inflammation reaction both under in vivo and in vitro conditions [235,239]. Because of these remarkable features, DLC coatings found various applications as coatings in many implant devices such as cardiovascular stents, heart valves, surgery needles, medical wires, contact lenses, etc. DLC coatings can also be used as protective coating in knee replacement because of their high corrosion resistance, hardness, and low wear rate. Generally, DLC films are used to reduce the frictional coefficient and offer better wear resistance [238]. Carbon based coatings and their significance in biomedical field are summarized in Table 6.

### 3.5. Calcium Phosphates

Calcium phosphate (CaP) ceramics are widely used as implants since they have a chemical composition similar to the inorganic composition of the bone. By controlling the surface properties such as roughness and porosity of CaP, one can regulate the biomineral formation and cell/protein adhesion. Bioactivity properties are varied depending on the type of calcium phosphates (HAp, tricalcium phosphate (TCP)) because of the differences in crystallinity, solubility, stability, ion release, and mechanical properties. At first, CaP coatings were deposited through the vapor phase process, but in recent years, biomimetic and solution-based methods were developed. Each synthesis approach has its own intrinsic properties, but in general, CaP based coatings are promising to improve implant longevity and biocompatibility. Many studies have been focused on the development of CaP ceramic coatings on metallic substrates to achieve the biological properties identical to a bulk and to enhance the implant durability and fixation [250,251,252,253].

Presently, atmospheric plasma spraying (APS) is currently employed to develop CaP coating on implant surfaces [2]. The CaP phases in the coatings exhibit higher solubility in an aqueous medium than HAp which is desirable for activating bone formation. However, faster dissolution reduces the stability and can cause loosening of the implant. A highly crystalline HAp phase dissolves in human physiological conditions at a lower rate which provides long-term stability of the implants. Thus, for the development of implants with required properties, one must control the purity and crystallinity of the coatings. CaP coatings with a denser microstructure lower the risk of delamination of the coating during in vivo tests with human body fluids. Coating surface roughness affects its dissolution and bone apposition and growth. Porous surfaces may enhance cell attachment or formation of the extra-cellular matrix, but the accumulation of macropores at the coating/substrate interface weakens the coating adhesion [254].

CaP in the form of HAp is widely used in implant applications due to its superior biological response. The HAp composition is Ca_10_(PO_4_)_6_(OH)_2_ (Ca/P = 1.67), which resembles the chemical composition of hard tissues such as bone and teeth [255]. Hence, HAp is considered as a primary candidate material due to its exceptional biological properties such as excellent biocompatibility, osteoconductivity, osteoinductivity, and bioactivity [256]. HAp coatings release calcium and phosphate ions and regulate the activation of osteoclasts and osteoblasts, facilitating bone regeneration [257]. The use of HAp ceramics enhances the regeneration of bones, improves osteoconductivity for bone growth, and promotes mineralization through ion release control and encapsulating growth factors. HAp ceramic coating enhances bone apposition in orthopedic implants through the formation of an extremely thin bonding layer with the existing bone. Due to such tissue bonding characteristics, Hap-based ceramics are considered as bioactive-based coatings. The continuous effort to improve the durability of the HAp ceramic coatings has led to development of high-quality HAp coatings and the development of Hap-based composite coatings.

**Figure 5:**
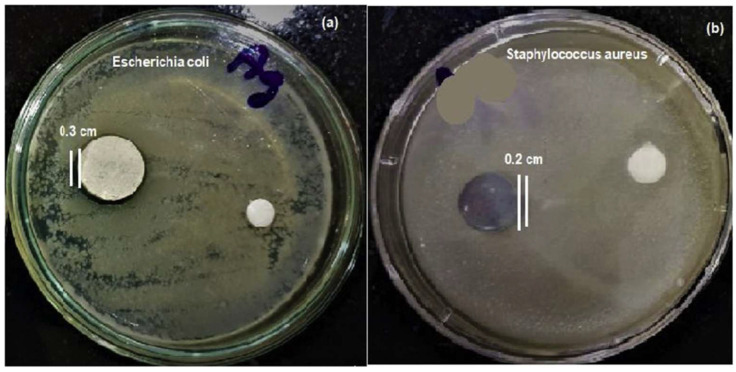
Demonstration of antimicrobial activity of HAp and Ag doped HAp composites against *E. coli* (Gram-negative) (**a**) and *S. aureus* (Gram-positive) (**b**) bacteria [261]. The photograph shows that Ag-doped HAp inhibits *S. aureus* bacteria more effectively than *E. coli*.

Highly porous or highly crystalline HAp coating shows poor adhesion to the substrate. Sankar et al. [258] studied the corrosion behavior of HAp coatings prepared by electrophoretic deposition (EPD) and the pulsed laser deposition (PLD) method. The corrosion results suggest that the HAp coatings show lower corrosion protection than the coatings prepared by the PLD method due to the formation of denser and pore-free coating [258]. Corrosion protection can also be enhanced by the addition of antimicrobial dopants. For example, Yugeswaran et al. [259] prepared HAp-TiO_2_ nanocomposite coatings by APS. The coating shows better corrosion performance in SBF medium than HAp coating without dopants due to its high compactness and the presence of TiO_2_ [259]. Silver (Ag) containing HAp coatings prepared by Trujillo et al. [260] show better antibacterial activity than HAp coating alone against *P. aeruginosa* and *S. epidermidis* pathogens due to the antibacterial activity of Ag. The antimicrobial activity of the Ag-doped HAp composite against *E. coli* and *S. aureus* was tested by Lett et al. [261]. The results indicated that the Ag-doped HAp composite has better inhibition of bacterial growth and shows a stronger ability against *S. aureus* bacteria to fight against toxic responses (Figure 5). The absence of Ag in the composite results in lower antibacterial activity of HAp composites. The variation in antibacterial activity was attributed to a thinner cell wall response of *S. aureus* (Figure 5b) to Ag ions than *E. coli* (Figure 5a) [261].

In biomedical implants, the major challenge for the performance of implants is bacterial invasion. During surgical operation, the bacteria may enter the surface of the implants through surgical equipment or cross contamination which form a biofilm. Once the surrounding implant is infected, the infection causes implant loosening. To overcome this issue, antimicrobial agents are used as dopants in ceramics, protecting implant material from bacterial invasion and improving their durability. Zinc doped HAp composites prepared by the sol-gel route and annealed at different temperatures (500 °C and 700 °C) show higher antimicrobial activity against *C. albicans* fungal cells and *S. aureus* bacteria [262].

Multiple doping of ions into HAp coatings was also attempted to improve their structural stability, partial dissolution, and biocompatibility. Wang et al. [263] prepared Sr and F^−^ doped hydroxyapatite and studied the properties of the coating. The addition of the dopant improves the structural stability of the HAp lattice and promotes osteogenic cell differentiation. Moreover, the addition of F^−^ ions potentially arrests the formation of *S. aureus*. Dopants such as Cu, Zn, Mg, Ag added to HAp enhance antibacterial activity and decrease the toxic effects towards the human body cells [264,265,266,267]. For example, Mg-doped HAp shows better osteoblast cell adhesion than pure HAp [268].

The differences in the thermal expansion coefficient of HAp and metallic alloys result in residual thermal stress. The stress accumulation increases with the increase in the coating thickness, which promotes cracking or delamination of the coating. For a thicker coating, the outer layer may detach from the implant, whereas a thin HAp coating can prematurely resorb during bone regeneration. Various HAp composites and their biological properties are summarized in Table 7.

### 3.6. Zirconia

Zirconia (ZrO_2_) is a ceramic material that can withstand high temperatures as well as higher stresses. It has widespread applications in dental implants and in the coatings on metallic implants to increase their corrosion resistance [280]. ZrO_2_ ceramics offer many advantages, including mechanical strength, chemical stability, biocompatibility, good aesthetics, and better wear resistance. Zirconia stabilized with yttria (YSZ) has been used as a dental implant due to its excellent mechanical strength and fracture toughness [281]. YSZ coatings show better hardness and scratch resistance than HAp coating [282]. Gobi Saravanan et al. [283] observed that the YSZ coated Ti substrates show improved hemocompatibility, activating blood platelets with pseudopods. In addition to that, superior in vitro biomineralization behavior was observed and documented through the weight gain on YSZ coating.

Zirconia stabilized with different weight fractions (0, 4, 10 wt. %) of yttria yields different phases (monoclinic, tetragonal, and cubic): zirconia ceramics with tailored mechanical properties and biocompatibility can be thus prepared. Attempts were made to deposit different phases of zirconia (*m*-ZrO_2_, *t*-ZrO_2_, and *c*-ZrO_2_) with the use of electron beam physical vapor deposition (EBPVD) [284]. All the coatings show lower surface roughness than coating prepared through the APS method and reduce pathogen bacterial invasion. Particularly, *t*-ZrO_2_ shows superior hardness over the other two zirconia phases. All the allotropes show better blood plasma protein adhesion and enhanced resistance to corrosion in comparison to uncoated medical grade stainless steel substrates in ABP solution.

Antibacterial activity of ZrO_2_ coating can be enhanced by the addition of Ag. Ag-ZrO_2_ composite coatings were prepared by Pradhaban et al. [285]. The results suggest that the coating shows antimicrobial activity against *E. coli*. Santos et al. [286] prepared glass ceramic composites with different concentrations of ZrO_2_ particles (0–50 vol. %) and carried out a ball-on-plate tribology test. ZrO_2_ glass ceramic composite (30 vol. % of ZrO_2_) shows optimal wear properties (coefficient of friction is 0.3) and is recommended for load-bearing applications. Bermi et al. [287] deposited YSZ coating through pulsed plasma deposition, and the tribological behavior of the coating in both dry and wet conditions was tested. YSZ coating deposited on a Ti6Al4V alloy ball sliding against the UHMWPE disk shows a reduction in wear rate (17% and 4% in dry and lubricated conditions) than uncoated alloy substrate.

**Figure 6:**
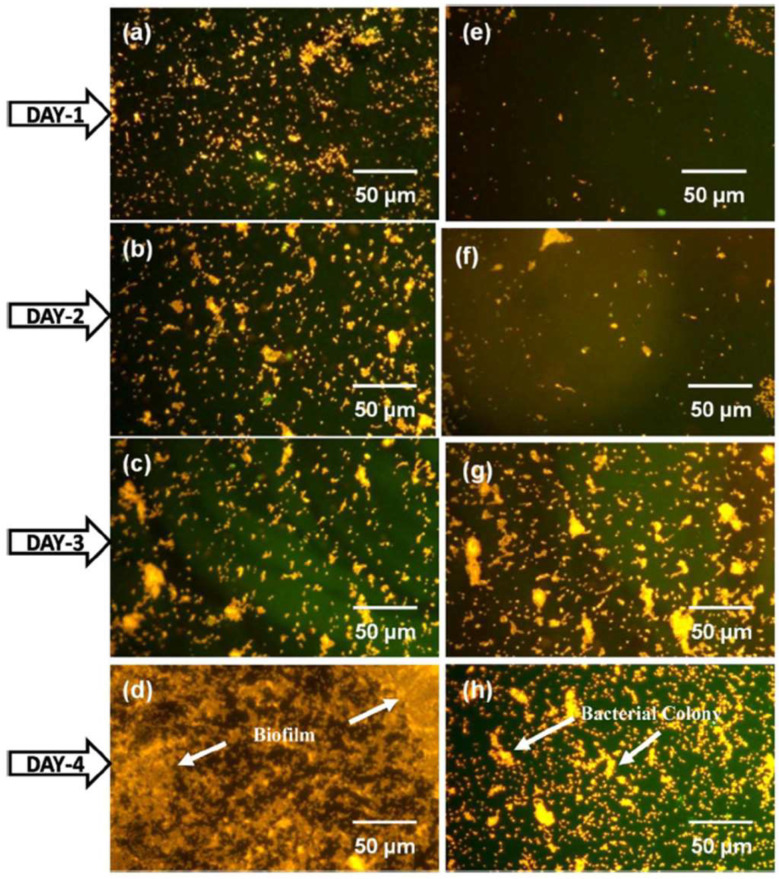
Epifluorescence microscopy analysis of P. aeruginosa bacterial invasion on 316L SS (**a**–**d**) and ZrO_2_ film (**e**–**h**) after 1, 2, 3, and 4 days incubation [288]. The used acridine orange staining shows orange color for live cells and green color for dead cells. The reduction in bacterial adhesion was seen on ZrO_2_ coated substrate compared to uncoated 316L SS.

Kaliaraj et al. [288] prepared zirconia coatings on a 316L SS substrate by electron beam physical vapor deposition (EBPVD), and a bacterial adhesion study with *P. aeruginosa* was carried out. Epifluorescence microscopy analysis of live/dead cells after incubation of 1, 2, 3, and 4 days showed a drastic reduction in bacterial adhesion on ZrO_2_ coatings, along with retardation in biofilm formation (Figure 6). This observation was attributed to the decrease in surface roughness obtained through coating deposition and the surface chemistry of ZrO_2_ that inhibits bacterial adhesion. Electrochemical impedance corrosion results show that ZrO_2_ exhibited superior corrosion resistance in the presence of H_2_O_2_ in an artificial blood plasma electrolyte solution. [288].

### 3.7. Bioactive Glass Coatings

Hench pioneered bioactive materials research and revolutionized the fields of bioactive materials and ceramics with his discovery of bioactive glass (45S5 composition), commercially known as Bioglass [289]. In the wake of Bioglass, various compositions and composites of bioactive glasses or silicates prepared both by melt quench and sol-gel techniques were investigated. Although bioactive glasses exhibit excellent bioactivity, because of their amorphous or semi-crystalline nature, they often fail as an implant material due to their poor mechanical strength. To overcome the shortage in mechanical properties, bioactive glasses are often composited with various metal oxides such as TiO_2_, Al_2_O_3_, ZrO_2_, and 2-D materials such as graphene and its derivatives (graphene oxide and reduced graphene oxide) [290]. These composites were reported to improve the corrosion resistance, antibacterial activity, and angiogenic properties of bioactive glass coatings without losing the bioactivity [291]. Similar to many ceramic materials, bioactive glasses can also be prepared in the form of particles of nano and micron size, as mesoporous particles, fibers, 3D scaffolds or monoliths, and thin films or coatings [292].

In this section, various types of coating technologies that can be used for the coating of bioactive glasses and their composites on different types of metals, alloys, and certain specific surfaces are discussed. One of the most simple and economical coating processes is the sol-gel dip-coating process. However, the coatings are often porous because of the solvent evaporation leading to poor corrosion resistance and mechanical properties. Nevertheless, this problem can be solved by incorporating metal oxides such as B_2_O_3_ as reported by Pinki Dey et al. [293]. According to their report, by replacing the silica weight percentage in the 45S5 system by 1% to 5 wt. %, they were able to decrease the porosity in the particles. Thermal spray coating, an industrial coating process, can also be employed for bioactive glass coating preparation. This process involves the coating of bioactive glasses as fine droplets or as plasma and sprayed over metal surfaces. Porous and non-porous layers with varying coating thicknesses can be achieved by the thermal spray process by tuning the deposition parameters such as velocity, size of the droplets, and temperature of the substrates [294].

Bioactive glasses can also be coated by physical deposition techniques such as radio-frequency magnetron sputtering (RF-MS) and pulsed laser deposition. In a recent study conducted by Qaisar Nawaz et al. [295], silver nanoclusters embedded in a silica matrix were deposited over the PEEK/BG layer using RF co-sputtering. They report a uniform 100 nm of the Ag-SiO_2_ layer that showed slower and sustained release of silver ions compared to the electrophoretically deposited coating. Although the physical deposition techniques are very robust and highly reproducible, their shortcoming is often the expensive experimental setup and precursors when compared to wet chemical sol-gel coating techniques. On the other hand, electrophoretic deposition (EPD) combines both the advantages and disadvantages of sol-gel coating and physical deposition methods. EPD is both a versatile and cost-effective method for coating ceramic materials on conducting surfaces.

**Figure 7:**
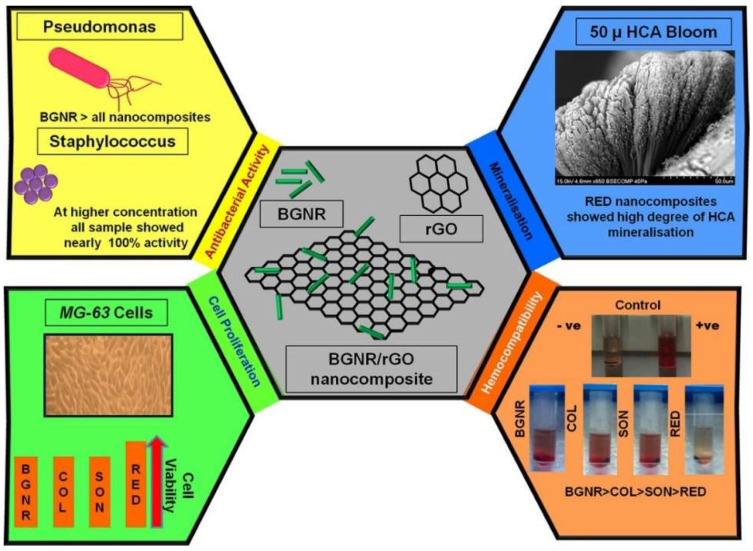
Schematics for HCA formation, antibacterial activity, hemocompatibility, and cell proliferation of bioactive glass rods (BGNR) and their composites with rGO (COL, SON, and RED) [297]. Figure also shows the bioactive behavior of the BGNR-rGO composites. It is noticed that the RED composites showed better HCA layer formation, cell proliferation, and hemocompatibility.

Ashokraja et al. [296] reported bioactivity in simulated body fluid (SBF) and reactive oxygen production using the XTT assay for reduced graphene oxide (rGO), sol-gel derived bioactive glass rods (BGNR) followed by different methods for developing composites of rGO and BGNR such as under constant stirring (COL), under constant sonication (SOL), and with a simultaneous reduction in graphene oxide-BGNR composites (RED). In their study, they report the role of pH changes in the sol-gel process facilitating one-dimensional rod-shaped bioactive glass formation, and their immersion studies exhibited a 50-micron thick HAp layer on the seventh day for rGO/BG composites [297]. Their work also reports that the different methods employed to prepare the composites influence the HCA formation, antibacterial efficacy, hemocompatibility, and cell proliferation as shown in Figure 7.

A recent comparative study reported results between pure BG and rGO/BG thin films deposited over the anodized surface of titanium by EPD. The deposited bioactive coatings (both pure and composites) were 2 µm thick and exhibited very good HAp formation in simulated body fluids along with super hydrophilicity in pure bioactive glass coatings [298]. Table 8 summarizes a brief list of bioactive glass coatings, their compositions, coating processes: important features are elucidated.

## 4. Summary and Future Directions

This paper reviews different biomaterials and explains their significant characteristics that influence their bioactivity. Bioimplant manufacturing involves an integrated process of selection of materials, design, fabrication, and surface modification through micro/nano texturing or coating application. Engineering of native metals by converting them into alloys yields desired properties and provides flexibility in designing the needs as per implant requirements. For a long-term application of bioimplants, surface characteristics and their biological functions are considered as key factors. Engineering the surface of the biomaterials by applying suitable coatings provides flexibility in tailoring the properties as per the requirements.

Bioceramic coatings hold great potential by tailoring the biological properties that suit our needs: the choice of the coating depends on the interaction between the cells with the coatings and substrates that are being used. Coatings on metallic implants are invaluable due to their functionality, biocompatibility, durability, and stability. Bioactive coatings are used to enhance the biological fixation between the bone and metallic implant despite their poor tribological and mechanical properties. Hence, they are often improved by developing composites with materials that possesses good mechanical strength. These improved coatings can be also used for durable load-bearing implants. All these properties lead to a better clinical success rate in long-term use in comparison to uncoated metallic implants. The bioactive ceramic coated biodegradable implants provide synergistic properties of both the implants and coating. Thus, these coatings find applications in cardiovascular stents, heart valves, orthopedic applications, tissue engineering, drug delivery, and biosensors. The current trends of ceramic coatings coated metallic implants are more focused on orthopedic applications.

Feasibility studies on complex structures, designing, fabrication of metallic alloys to form complex shapes without losing mechanical properties and surface integrity are a challenging task and should be attempted. The degradation mechanism of coatings on metallic implants changes in the human body environment. Moreover, lattice mismatch and the accumulation of residual stress cause degradation of the implant after implantation. Thus, there is a need to develop mathematical models for the prediction of degradation mechanisms. Another approach to reducing the residual stress is to deposit a functionally graded multi-layered or nanocomposite coating with multifunctional properties.

## Figures and Tables

**Table 1:** Ceramic coatings used for biomedical applications [18].

Coatings	Applications	Advantages
Oxides (TiO_2_, ZrO_2_)	Oral implant application Maxillofacial reconstruction Ophthalmic implants	Good regenerative capability Corrosion resistance Antibacterial activities
Nitrides (TiN, ZrN, TiCN, ZrCN, TiAlN) and Oxynitrides (TiON, ZrON)	Dental implants Fracture fixation devices Components of joint endoprostheses	Resistance to corrosion Low frictional coefficient Better adhesion to the substrates
Carbon Based Coatings (a-C, DLC, NCD, carbides, and carbontirides)	Artificial heart valves Orthopedic fixation devices Sensors Artificial ligaments	Low frictional coefficient Excellent biocompatibility High blood compatibility Hydrophobicity
Calcium phosphates (CaP, HAp) and bioactive glass	Spinal implants Orthopedic implants Maxillofacial reconstruction Skull plates	High osteointegration capability Excellent biocompatibility Bioactivity

**Table 2:** The pros and cons of various biomaterials used in the biomedical industry [118].

Materials	Advantages	Disadvantages	Applications
Polymers	Good performance in cyclic load applications, degrade completely over time.	Different cytotoxicity mechanism, inflammatory reactions, bone degradation, show higher corrosion rate.	Bearing surfaces [119]
Ceramics	Zero risk of transmitting diseases/immunogenicity, compression force resistance, corrosion resistance.	Low mechanical properties, high stress-shielding effects, lower rate of biodegradation, fracture toughness is poor.	Bearing surfaces
Stainless Steels	Better mechanical strength, high ductility, flexibility in bending, low manufacturing cost.	High stress-shielding effects, low resistance to corrosion, less osseointegration, biocompatibility issue.	Bone plates, pins, nails, screws, threads, steel threads, and sutures
Co-Cr based alloys	High strength, ductility, elastic modulus, stiffness, and density.	Higher modulus than bones, stress-shielding effects, not ideal for bearing surfaces in a joint, low frictional properties.	Orthopedic implants for knee, ankle, hip, shoulder, and fracture fixation devices
Titanium and its alloys	Good corrosion resistance, light weight, low density, good mechanical strength.	Poor tribological performance, high frictional coefficient, adhesive wear, and low abrasion resistance.	Total knee, hip replacement, bone plates, and screws for fixation and maxillofacial applications
Mg and its alloys	Low Young’s modulus, no stress shielding, biodegradable.	Biocompatibility issue, corrosion resistance, low mechanical integrity.	Mesh cage for segmental defects in bone, 3D scaffold design for better bone regeneration

**Table 3:** Methods and properties of PEEK-based composite coatings.

S. No.	Coatings	Deposition Method	Significance	Ref.
1	PEEK coating on Ti alloy (Ti-13Nb-13Zr)	Electrophoretic deposition (EPD)	Excellent wear resistance Very good adhesion Low frictional coefficients	[136]
2	HAp/PEEK composite coating on PEEK substrate	Cold Spray coating	Better biocompatibility and osseointgration for clinical applications	[149]
3	SiC/PEEK composite coating on SS	electrostatic spray coating method	Scratch resistance Hardness increases	[150]
4	h-BN/bioactive glass/PEEK coating on SS 316L	Electrophoretic deposition (EPD)	Good adhesion strength Wetting behavior	[137]
5	PEEK/HAp on 316L SS	Electrophoretic deposition (EPD)	Good antibacterial activity	[151]
6	PEEK coating on Ti implant	Thermal spraying	Improved stability and fracture resistance Abrasion resistance	[152]
7	PEEK/ Bioglass composite coating on PEEK substrates	Cold gas spray	Better wear resistance Biomechanical performance	[143]
8	ZrO_2_/PEEK coating on Ti6Al4V substrates	Thermal spraying	Improved wettability Blood compatibility Great potential for medical applications	[153]
9	Al_2_O_3_/PEEK, SiO_2_/PEEK coatings on Ti6Al4V substrates	Thermal spraying	High hardness Optimum tribological properties Potential candidate for bearing material	[154]

**Table 5:** Recent work on binary, ternary, and quaternary systems of transition metal nitride and carbide coatings for implant applications.

S. No.	Coatings	Deposition Method	Significance	Refs.
1	Nano-TiN coating on Ti-6A1-4V	Magnetron sputtering	Enhanced hardness and anti-wear resistance, good hemocompatibility, and biocompatibility	[207]
2	TiN coating on Ti alloy	Cathodic arc deposition	Better corrosion protection Low wear rate Reduced coefficient of friction	[208,209]
3	TiON coating on 316L SS	Magnetron sputtering	Better adhesion Good resistance to corrosion	[210]
4	TiON coating on Ti substrates	Magnetron sputtering	Better biological activity Highly biocompatible	[211]
5	TiCN coating on Ni-Cr alloy	Magnetron sputtering	Good adhesion of fibroblasts Less cytotoxic	[212]
6	TiZr/a-C coatings on Ti substrate	Cathodic arc deposition	Good compatibility with human skin fibroblast cells Good human skin fibroblast cell viability	[213]
7	TiZrCN, TiNbCN, and TiSiCN coatings on steel substrates	Cathodic arc deposition	Better adhesion to the substrate Corrosion resistance	[214]
8	TiAlN coating	Multi arc ion plating technique	Better tribological performance	[215]
9	Nanolayer CrAlN/TiSiN coating on steel substrates	Magnetron sputtering	Excellent tribological performance	[216]
10	TiCN/TiAlN and TiAlN/TiCN bilayer nitride coatings on cemented carbide substrates	Cathodic arc deposition	Higher hardness High scratch resistance	[217]
11	CoCrMoC/CrN and CrN/CoCrMoC coatings on medical grade SS substrates	Magnetron sputtering	Better tribo-corrosion behavior	[218]

**Table 6:** Different carbon coatings and their properties.

S. No.	Coatings	Deposition Method	Significance	Ref.
1	DLC on Ti alloy	Plasma immersion ion deposition (PIID)	Improvement in tribo-corrosion behavior	[240]
2	Si-DLC on Polyethylene (PE) substrates	Plasma and laser-based processing methods	Improvement in hydrophobicity, lubricity, and electrical conductivity	[241]
3	Carbon coatings on X39CR13 and 316LVM steels	Magnetron sputtering	Improved adhesion and wettability properties	[242]
4	Amorphous carbon/diamond-like carbon (a-C:H) coatings on PEEK substrate	Plasma enhanced chemical vapor deposition	No toxicity issues and better biological performance	[243]
5	DLC with Zr interlayers on Ti alloy	Magnetron sputtering	Reduced coefficient of friction	[244]
6	Si-DLC Coatings on Ti alloy	Magnetron sputtering	High level of biocompatibility due to the presence of Si	[245]
7	a-C:H coating on Co-Cr alloy	PVD/PE-CVD	Excellent mechanical properties, high hardness, and elastic modulus	[246]
8	Si doped DLC on Ti alloy	Magnetron sputtering	Reduced microbial colonization of *E. coli*	[247]
9	DLC on stainless steel	Pulsed DC PE-CVD	Improved biocompatibility and corrosion resistance	[248]
10	DLC with TiO_2_ on stainless steel	PE-CVD	Better biocompatibility and antimicrobial activity	[249]

**Table 7:** Hydroxyapatite and its composites’ coatings for implant applications.

S. No.	Coatings	Deposition Method	Significance	Ref.
	HAp nanowire coating on glass substrate	Solvothermal method	Excellent apatite-forming ability	[269]
	Fe doped HAp on Si substrate	Co-precipitation method	Promote better proliferation and adhesion of the osteoblast cells	[270]
	Ce doped HAp/collagen coating on Ti surface	Biomimetic method	Better antibacterial efficacy against *Escherichia coli* and *Staphylococcus aureus* bacteria than HAp coating	[271]
	Si substituted HAp coating on Ti substrate	Precipitation method	Favorable regeneration of crystalline Si-HA layer	[272]
	HAp/CaSiO_3_/Chitosan Porous coating on Ti substrate	EPD	Improved bioactivity and biocompatibility	[273]
	Bioactive glass/HAp coatings on Ti substrate	Pulsed laser deposition	Significant bioactivity, cytocompatibility, and hemocompatibility	[274]
	PyC/SiC/HAp coating on carbon fibers	Chemical vapor deposition/pulsed electrochemical deposition	Excellent corrosion resistance, induces the nucleation process and growth of bone-like apatite	[275]
	PEEK/HAp composite coating on 316L SS substrate	Electrophoretic deposition	Enhanced in vitro bioactivity	[148]
	Ag/HAp coating on Ti substrate	Sol-gel route	Enhanced antibacterial activity and better corrosion protection	[276]
	TiO_2_/HAp coating on Ti substrate	High velocity oxy fuel (HVOF) method	Improved corrosion resistance	[277]
	B_2_O_3_/Al_2_O_3_/HAp coating on Ti substrate	High velocity oxy fuel (HVOF) method	Improved adhesion strength	[278]
	TiO_2_/HAp nanocomposite coating on 316L SS substrate	Electrophoretic deposition	Excellent corrosion protection under SBF medium	[279]

**Table 8:** Composition, the substrate used, coating process, and their salient features of bioactive glasses.

S. No.	Coatings	Substrate	Deposition Method	Significance	Ref.
1	Titanium, HAp, Bioactive glass wt.% (57–60 SiO_2_, 21–24 CaO, 9–11 Na2O, 2–3P_2_O_5_, 0.5–1.5 TiO_2_, and 2–3B_2_O_3_)	Ti-alloy—Ti6Al4 V	Laser engineered net shaping	Improved hardness and wear resistance	[299]
2	58S Bioactive glass (molar composition of 35% CaO, 60% SiO_2_, and 5% P_2_O_5_) seeded in HAp	Commercial AISI 316L SS	Cold uniaxial pressing	Seeding of HAp increased the hardness as well as apatite layer formation	[300]
3	Bioglass with silver nanoparticles and Chitosan	Ti-alloy—Ti6Al4 V	Electrophoretic deposition	Increased coating uniformity and nanoscale roughness for bioactivity	[301]
4	(1) 65% SiO_2_, 5% P_2_O_5_, and 30% CaO, (2) 45% SiO_2_, 5% P_2_O_5_, and 50% CaO	Carbon foam	Dip Coating	Compact and dense coating is reported in 65% rather than 45% SiO_2_	[302]
5	Manganese modified Bioglass/alginate	316L SS	Electrophoretic deposition	Increase in manganese improves the corrosion resistance in SBF	[303]
6	Bioglass composite with chitosan and iron oxide nanoparticles	Ti-alloy—Ti–13Nb–13Zr	Electrophoretic deposition	Better corrosion resistance, coating adhesion, and hydrophilicity	[304]
7	Silver incorporated HAp and Bioglass	Nickel titanium alloy	Dip coating	Increased corrosion resistance and coating adhesion	[305]
8	Bioglass	AISI 304L SS	APS	Improved mechanical strength and corrosion resistance	[306]
9	Bioglass, TiO_2_, Al_2_O_3_, and Hap composite with PMMA	Stainless steel 304	Dip coating	PMMA-TiO_2_ coating exhibited higher corrosion resistance than other composites coatings	[307]
10	58S bioactive glass-gelatin-polycaprolactone composite	316L SS	Electrospinning	Increase in bioactive glass weight % improved surface Roughness and adhesion strength, exhibited good corrosion resistance, apatite formation and cell viability	[308]
11	58S Bioactive glass	Vitallium alloy	Dip coating	Decreased porosity and increased bioactivity	[309]
12	Bioglass	Ti6Al4V alloy	Electrophoretic deposition	Scratch resistance, hardness, and coating bonding strength	[310]
13	HAp-Bioglass-Iron oxide composite	Ti-alloy—Ti-13Nb-13Zr	Electrophoretic deposition	Corrosion resistance and non-toxic effects	[311]
14	Reduced graphene oxide—Bioglass sol-gel composite	Grade 2 titanium	Electrophoretic deposition	rGO facilitated low hemolysis and improved cell proliferation	[298]

## Data Availability

Not Applicable.
